# Severe eosinophilia and subsequent dermatologic immune‐related adverse event with squamous cell carcinoma antigen elevation induced by nivolumab and ipilimumab

**DOI:** 10.1002/rcr2.1037

**Published:** 2022-09-09

**Authors:** Takeshi Matsumoto, Akiko Kaneko, Yusuke Kusakabe, Emi Nakayama, Ayaka Tanaka, Naoki Yamamoto, Kensaku Aihara, Shinpachi Yamaoka, Michiaki Mishima

**Affiliations:** ^1^ Department of Respiratory Medicine Saiseikai‐Noe Hospital Osaka Japan

**Keywords:** Immune‐related adverse event, ipilimumab, nivolumab, severe eosinophilia, squamous cell carcinoma antigen.

## Abstract

Immune checkpoint inhibitors (ICIs) for malignant lesions are associated with immune‐related adverse events (irAEs), but reports about severe eosinophilia induced by ICIs are scarce. A 73‐year‐old man with lung squamous cell carcinoma was treated by chemotherapy (carboplatin plus paclitaxel) and ICIs (nivolumab plus ipilimumab). After two cycles of chemotherapy, the ICIs were continued. After 5 months, the eosinophilia, which had exceeded 5000/μl, increasingly deteriorated, and the only detected irAE was a grade 1 rash. Under continuation of the ICIs, although the eosinophilia decreased, a grade 3 rash and severe pruritis subsequently appeared. Squamous cell carcinoma antigen (SCCA) was steeply increased simultaneously. A complete response had been achieved, and oral prednisolone markedly improved the rash, pruritis, and eosinophilia. Clinicians should be aware that precedent severe eosinophilia and subsequent severe irAE could occur in patients treated by nivolumab and ipilimumab, and SCCA elevation could be associated with dermatologic irAE.

## Graphical abstract

We report the first case of severe eosinophilia induced by nivolumab and ipilimumab followed by a severe dermatologic immune‐related adverse events (irAEs). This report highlights the fact that clinicians should be aware that precedent severe eosinophilia and subsequent severe irAE could occur in patients treated by nivolumab and ipilimumab, and squamous cell carcinoma antigen elevation could be associated with dermatologic irAE.

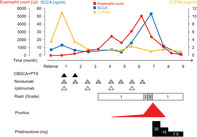

## INTRODUCTION

Lung cancer is very common and is the leading cause of cancer‐related death.^1^ The use of an immune checkpoint inhibitor (ICI) has proven to be effective for patients with many different types of cancers including lung cancer.^2^ However, ICI treatment is associated with immune‐related adverse events (irAEs), including dermatologic, gastrointestinal and rarely, hematologic events, such as eosinophilia.^3^

Eosinophilia is defined as an absolute eosinophil count of more than 500 cells/μl, and eosinophilia >5000 cells/μl is considered to be severe.^4^ Previously, in a large cohort of 948 patients, 35 (3.7%) were reported to have hematologic irAEs related to ICIs. Neutropenia, autoimmune hemolytic anaemia, and immune thrombocytopenia were the most common types of hematologic irAEs.^5^ An additional study noted that the incidence of eosinophilia with ICIs appears to be rare (2.8%), and the median eosinophil count peak has been around 1000 cells/μl [range 600–5600].^6^ Reports about severe eosinophilia induced by ICIs are surprisingly scarce. In addition, other irAEs related to eosinophilia usually occur before or simultaneously with the peak of the eosinophilia, though studies that report the timing are limited.^7^

We herein report a first case of severe eosinophilia induced by nivolumab (anti‐programmed death 1 antibody) and ipilimumab (anti‐cytotoxic T‐lymphocyte antigen 4 antibody) followed by a severe dermatologic irAE. We also provide a literature review to clarify the characteristics of eosinophilia induced by ICIs.

### Case report

**FIGURE 1:**
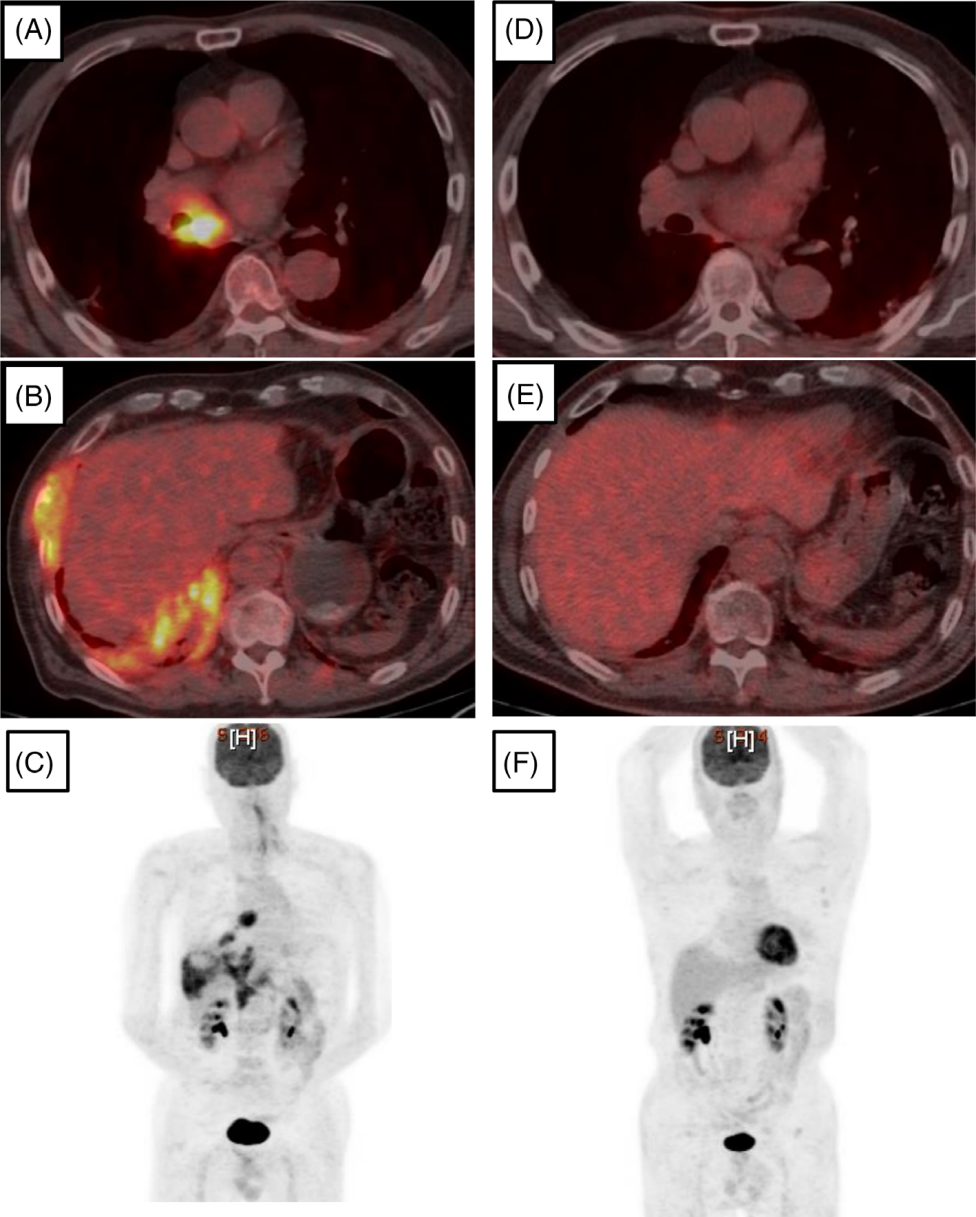
Radiological appearance of the PET scans at the time of the referral and 7 months after the treatment. (A–C) PET scan at the time of referral, showing high uptake of FDG in the mediastinal lymph nodes (SUV max = 11.1) and right pleural thickening (SUV max = 9.2); (D–F) PET scan 7 months after the treatment, showing no abnormal uptake of FDG. FDG, fluorodeoxyglucose; SUV, standardized uptake value; PET, positron emission tomography

A 73‐year‐old man was referred to our hospital with right‐sided chest pain and an abnormal shadow on his chest X‐ray. The patient has a history of hypertension and dyslipidemia and is an active smoker. No allergies to drugs or foods were reported. A chest X‐ray revealed consolidation in the right lower lung field. A computed tomography (CT) scan of the lungs showed a mass in the right lower lobe and right pleural thickening with mediastinal lymph node swelling. A positron emission tomography (PET) scan showed high uptake of fluorodeoxyglucose in the right‐sided mass (standardized uptake value [SUV] max = 6.8), in the mediastinal lymph nodes (SUV max = 11.1), and in the right pleural thickening (SUV max = 9.2) (Figure 1A–C). Laboratory testing revealed the following values: white blood cell count, 13,500/mm^3^ with 0.1% eosinophils; cytokeratin 19 fragment (CYFRA), 3.6 ng/ml; and squamous cell carcinoma antigen (SCCA), 7.1 ng/ml. Histological examination of a bronchoscopic biopsy of the right mass revealed squamous cell carcinoma. The patient was diagnosed with lung squamous cell carcinoma (cT1cN2M1b, stage 4A). The value of anti‐programmed death ligand 1 antibody immunohistochemistry 22C3 pharmDx was <1%.

**FIGURE 2:**
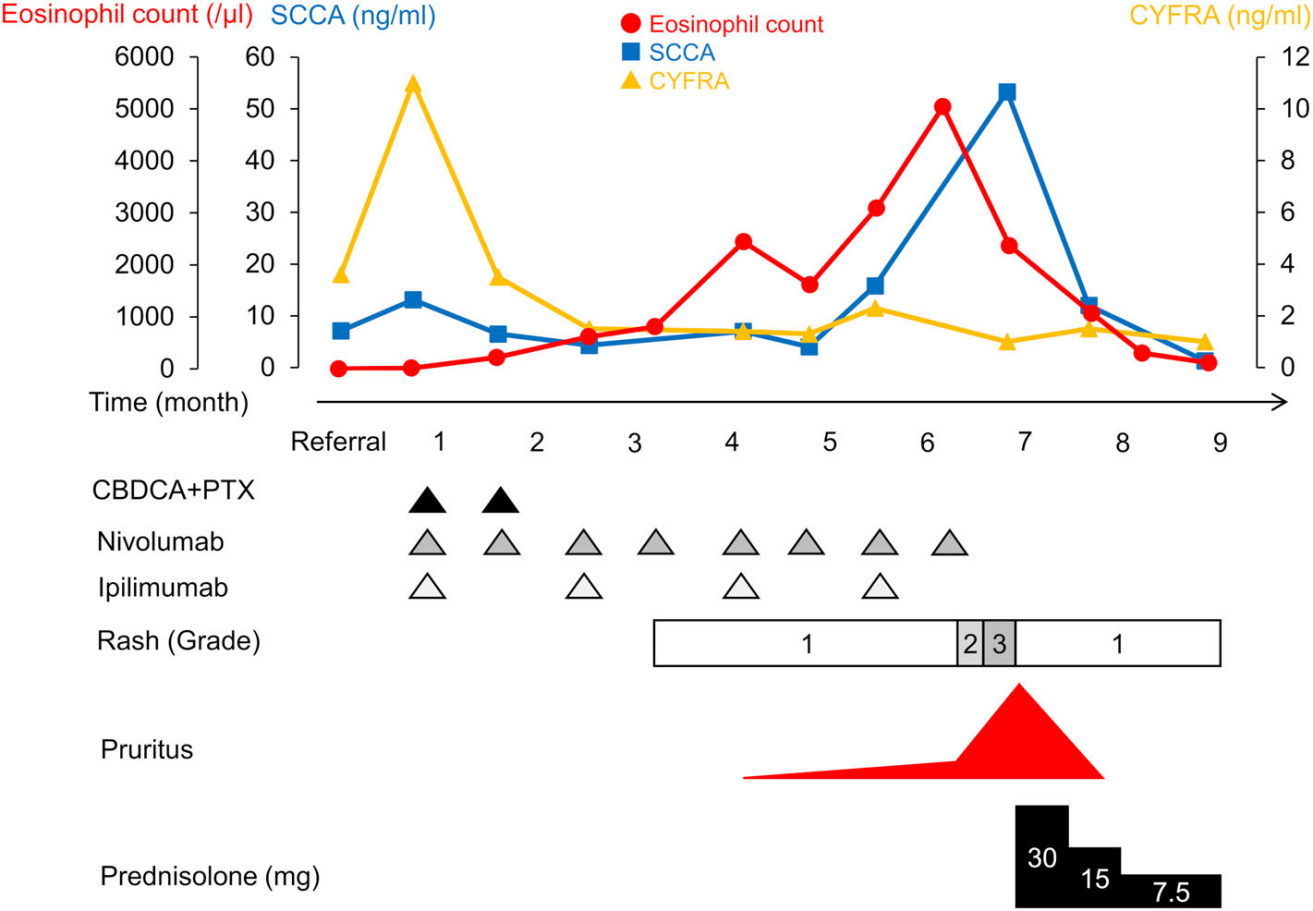
Clinical course in this case. CBDCA, carboplatin; CYFRA, cytokeratin 19 fragment; PTX, paclitaxel; SCCA, squamous cell carcinoma antigen

Chemotherapy (carboplatin area under the curve 4, day 1; plus, paclitaxel 160 mg/m^2^, day 1) combined with ICIs (nivolumab 360 mg/body, day 1; plus, ipilimumab 1 mg/kg, day1) was commenced, resulting in shrinkage of the mass. After two cycles of chemotherapy, ICIs were continued as maintenance therapy. Two months following the beginning of treatment, a grade 1 erythematous rash (according to the Common Terminology Criteria for Adverse Events, version 5.0) appeared, and mild pruritus appeared 3 months later, but they were well controlled by topical medications. Considering clinical course and no other added drugs, the rash was thought as irAE. At the same time, mild eosinophilia occurred, which was thought to be induced by ICIs because there were no apparent findings of infection including parasites or atypical cells in peripheral blood. However, after 5 months, the patient's eosinophil count drastically increased, exceeding 5000/μl; however, only a grade 1 rash was detected as an irAE. Per request of the patient, the ICIs were continued. Although the eosinophil count decreased, a grade 3 erythematous rash and severe pruritus subsequently appeared. SCCA steeply increased, while CYFRA decreased and remained within normal limits after the initiation of chemotherapy. A PET scan showed a complete response (CR) (Figure 1D–F), and an upper gastric endoscopy revealed no abnormality. Therefore, the elevation of SCCA was thought to have resulted from the severe rash. Prednisolone was commenced at 30 mg per day, which markedly improved the rash, pruritis, and eosinophilia. The prednisolone has since been tapered, and there was neither recurrence of the cancer nor of an irAE following 3 months of cessation of chemotherapy (Figure 2).

## DISCUSSION

We reported a case of severe eosinophilia induced by nivolumab and ipilimumab followed by severe dermatologic irAE. In this case, the timing of the severe irAE was late compared to the appearance of the severe eosinophilia, and SCCA increased according to the increase of the severity of the dermatologic irAE, rather than to the growth of the tumour. Use of a systemic steroid rapidly improved the eosinophilia and irAE, and a CR was achieved.

Although irAEs are common during treatment with ICIs, eosinophilia is rare, and especially, severe eosinophilia is scarce. To date, the reports about severe eosinophilia facilitated by ICIs were mostly case reports,^8^, ^9^, ^10^ with only two large studies focused on patients with eosinophilia. One reported the prevalence of eosinophilia as 2.8% in 909 patients^6^; however, the patients who received a combination of ICIs were excluded, and the prevalence of severe eosinophilia was not shown. Another reported the prevalence of moderate‐to‐severe eosinophilia as 37 in 1546 patients (2.4%) and severe eosinophilia as 10 patients (0.6%).^11^ To our knowledge, for a combination therapy of nivolumab and ipilimumab, there has been no report of severe eosinophilia to date. Lung cancer, in particular, may be associated with an increased risk of eosinophilia when treated with ICIs compared to other cancers.^12^ In addition, the combination of ICIs, or combination with paclitaxel chemotherapy may increase eosinophilia compared to the monotherapy of ICI.^13^, ^14^ Although a high eosinophil count alone may not necessarily serve as an index of severity,^11^ clinicians should account for the possibility of the development of severe eosinophilia in patients treated with nivolumab and ipilimumab, which are increasingly utilized in patients with lung cancer, as severe eosinophilia could be the sign of severe irAE.

Is eosinophilia harmful? Actually, eosinophilia is reported to be a prognostic factor in patients treated by ICIs for melanoma^15^, ^16^ and lung cancer.^6^, ^12^ The assumed mechanisms were T‐cell recruitment and polarization using chemokines, induction of dendritic cell activation and recruitment, enhancement of tumour immune surveillance, and direct antitumor effects.^8^, ^17^ In addition, tumour eosinophil infiltrating cells may be involved in tumour control through secretion of chemoattractants by eosinophils recruiting CD8‐positive T‐cell and macrophages, which could contribute to the favourable results.^18^ In this case, a CR was achieved. On the other hand, there have been contradicting reports claiming eosinophils were not associated with benefits.^19^, ^20^ The clinical value of eosinophilia as a biomarker may require additional investigation.

Reports addressing the association between the onset of an irAE and eosinophil counts are scarce. In a study of lung cancer, eosinophilia was observed more than a month before the onset of symptoms of adrenal insufficiency; however, the timing of peak eosinophil count was almost the same as the timing of symptom onset.^7^ We usually experience the coincidence of an increase in eosinophil counts and irAE. The median peak of eosinophil count was achieved at a median of 6.4 months,^6^ or 15 weeks,^11^ in the former two studies. In this case, severe eosinophilia was seen with a grade 1 irAE after 5 months. Thereafter, there was mild improvement in the eosinophilia; however, a more severe irAE occurred. Cumulative eosinophilia might cause this delayed severe irAE. Although the precise mechanism remains to be elucidated, clinicians should be aware of delayed irAEs after an increase in eosinophil count.

At first, CYFRA and SCCA were decreased in response to chemotherapy, which means they were favourable markers of tumour growth; however, SCCA unnaturally elevated, although CYFRA remained decreased. SCCA could elevate in malignant and non‐malignant squamous lesions.^21^ Although the exact nature and pathophysiology of SCCA have not yet been fully clarified, it is speculated that the antibody recognizes an epitope on one or more of the small cytokeratins. The cytoplasmatic staining pattern and tissue localization, the absence in normal epidermis in contrast to the expression in hyperproliferative and neoplastic epithelium, the approximate molecular weight and the demonstration of an acidic and a basic subfamily are all arguments in favour of this hypothesis. Therefore, the existence of an inflammatory skin disease or a hyperkeratotic skin disease with an inflammatory component may be associated with the elevation of SCCA.^22^ SCCA increase was eventually considered to be associated with dermatologic irAE in this case. Therefore, it may be useful to monitor several tumour markers for the detection of severe dermatologic irAE as well as tumour growth. To our knowledge, a report addressing the association between SCCA elevation and dermatologic irAE has not been found to date; therefore, more studies are needed to further clarify this relationship.

In conclusion, clinicians should be aware that precedent severe eosinophilia and subsequent severe irAE could occur in patients treated by nivolumab and ipilimumab, and it may be useful to monitor several tumour markers (including SCCA and CYFRA) for the detection of severe dermatologic irAE as well as tumour growth.

## AUTHOR CONTRIBUTIONS

Takeshi Matsumoto drafted the manuscript. All authors interpreted the data, critically revised the manuscript for intellectual content, and approved the final version of manuscript.

## CONFLICT OF INTEREST

None declared.

## ETHICS STATEMENT

The authors declare that appropriate written informed consent was obtained for the publication of this manuscript and accompanying images.

## Data Availability

The data that support the findings of this study are available on request from the corresponding author. The data are not publicly available due to privacy or ethical restrictions.
